# Pricklier with the proper predator? Predator‐induced small‐scale changes of spinescence in *Daphnia*

**DOI:** 10.1002/ece3.8346

**Published:** 2021-11-17

**Authors:** Patricia Diel, Max Rabus, Christian Laforsch

**Affiliations:** ^1^ Department of Animal Ecology 1 University of Bayreuth Bayreuth Germany

**Keywords:** *Daphnia*, inducible defenses, phenotypic plasticity, predation, predator–prey interaction, spinules

## Abstract

Phenotypic plasticity in defensive traits is a common response of prey organisms to variable and unpredictable predation regimes and risks. Cladocerans of the genus *Daphnia* are keystone species in the food web of lentic freshwater bodies and are well known for their ability to express a large variety of inducible morphological defenses in response to invertebrate and vertebrate predator kairomones. The developed defenses render the daphnids less susceptible to predation. So far, primarily large‐scale morphological defenses, like helmets, crests, and tail‐spines, have been documented. However, less is known on whether the tiny spinules, rather inconspicuous traits which cover many *Daphnia*’s dorsal and ventral carapace margins, respond to predator kairomones, as well. For this reason, we investigated two *Daphnia* species (*D*. *magna* and *D. longicephala*) concerning their predator kairomone‐induced changes in dorsal and ventral spinules. Since these small, inconspicuous traits may only act as a defense against predatory invertebrates, with fine‐structured catching apparatuses, and not against vertebrate predators, we exposed them to both, an invertebrate (*Triops cancriformis* or *Notontecta maculata*) and a vertebrate predator (*Leucaspius delineatus*). Our results show that the length of these spinules as well as spinules‐covered areas vary, likely depending on the predator the prey is exposed to. We further present first indications of a *Daphnia* species‐specific elongation of the spinules and an increase of the spinules‐bearing areas. Although we cannot exclude that spinescence is altered because it is developmentally connected to changes in body shape in general, our results suggest that the inducible alterations to the spinule length and spinules‐covered areas disclose another level of predator‐induced changes in two common *Daphnia* species. The predator‐induced changes on this level together with the large‐scale and ultrastructural defensive traits may act as the overall morphological defense, adjusted to specific predator regimes in nature.

## Graphical abstract

We investigated two Daphnia species (*D*. *magna* and *D. longicephala*) concerning their predator kairomone‐induced changes of the dorsal and ventral spinules. Since these small, inconspicuous traits may only act as a defense against predatory invertebrates, with fine‐structured catching apparatuses, and not against vertebrate predators, we exposed them to both, an invertebrate (*Triops cancriformis* or *Notontecta* *maculata*) and a vertebrate predator (*Leucaspius delineatus*). Our study discovers indications of predator‐dependent, alongside *Daphnia* clone‐specific, defensive increases in spinescence.

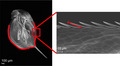

## 1 INTRODUCTION

The interaction of predators and prey has led to the evolution of various defenses in prey organisms. Such defenses can be differentiated into constitutive and inducible defenses (Agrawal et al., 1999; Harvell, 1990; Harvell & Tollrian, 1999). While constitutive defenses evolved in the rather permanent presence of natural enemies, inducible defenses represent a prey's reaction to an inconsistent, yet at times strong predation pressure (Harvell, 1990; Harvell & Tollrian, 1999). Additionally, inducible defenses involve costs, which top the benefit of the defenses at times of no predation threat (Harvell, 1990; Harvell & Tollrian, 1999; Riessen, 2012). Inducible defenses can be found across various taxa. The freshwater crustacean genus *Daphnia* provides many well‐studied examples of inducible defenses against vertebrate and/or invertebrate aquatic predators, reviewed by Riessen and Gilbert (2019) and Diel et al. (2020). For these model organisms, mechanical and chemical cues can trigger the expression of inducible defenses (Laforsch et al., 2006; Laforsch & Tollrian, 2004a). The predator‐related chemical cues entail so‐called kairomones and alarm cues. Kairomones are interspecific semiochemicals, which the predator releases unintentionally and which are beneficial for the receiver but not for the sender (Brown et al., 1970; Harvell & Tollrian, 1999; Weiss et al., 2018). Alarm cues, on the other hand, are chemical cues, which derive from wounded prey and were shown to have a defense‐inducing effect between conspecifics of *Daphnia* species, and in a more moderate form even across *Daphnia* species (Laforsch et al., 2006). In the past, the majority of studies on inducible morphological defenses of *Daphnia* focused on rather prominent defenses like the development of helmets, for example, in *D. cucullata* (Laforsch & Tollrian, 2004b), as well as the expression of large expansions of the dorsal head region in the form of a crest, for example, in the *D. carinata* King complex (Grant & Bayly, 1981), the elongation of tail‐spines, for example, in *D. lumholtzi* (Tollrian, 1994), and the change of the entire body appearance, that is, abandonment of the bilateral symmetry in *D. barbata* (Herzog et al., 2016) in response to kairomones. Furthermore, a few studies also focused on ultrastructural defensive changes to the structure of the carapace accompanying the large‐scale defenses in *Daphnia* (Dodson, 1984; Kruppert et al., 2017; Laforsch et al., 2004; Rabus et al., 2013). In response to larvae of the phantom midge *Chaoborus* sp., some *Daphnia* species were also found to develop small, outwardly positioned so called neckteeth, for example, *D. pulex* (Krueger & Dodson, 1981; Tollrian, 1993, 1995) and *D. curvirostris* (Juračka et al., 2011), which reduce the mortality risk in the most threatened prey instars (Tollrian, 1995). While most *Daphnia* species carry small spinules along their dorsal and ventral carapace rims permanently (Benzie, 2005), some studies indicate that these traits respond to the presence of predators as well (Fryer, 1991; Hebert & Grewe, 1985; Herzog & Laforsch, 2013; Ritschar et al., 2020).

However, it is not known if the predator‐induced alteration in spinescence is a general response to predator stress in *Daphnia* or if the response is species‐ or predator‐specific. With this study, we sought to investigate whether two previously thoroughly studied *Daphnia* species (i.e., *D*. *magna* and *D. longicephala*) express an enhanced spinescence, that is, changes concerning the spinule length and/or spinules‐bearing area (hereafter called SBA) along the carapace rims, along their carapace margins in reaction to predator kairomones released by the tadpole shrimp *Triops cancriformis* or the backswimmer *Notonecta maculata*, respectively. We hypothesized that a defensive increase in spinescence would occur in both species since previous studies demonstrated the expression, as well as the effectiveness, of morphological comparatively small (Rabus et al., 2013; Tollrian, 1995) as well as large‐scale defenses in response to predatory invertebrates (Laforsch et al., 2006; Rabus & Laforsch, 2011; Rabus et al., 2012; Weiss et al., 2015). However, we expected the induced changes in spinescence to differ with respect to the mean spinule lengths and the relative lengths of the SBAs between the two species, as do the macrodefenses, which could potentially be related to the various techniques of catching, handling, and ingesting of prey, performed by different predator taxa. Previous studies on predator‐induced alterations in spinules focused on invertebrate predators only (Hebert & Grewe, 1985; Herzog & Laforsch, 2013; Ritschar et al., 2020). Given that these small‐scale traits may only interact with the catching apparatuses, or mouthparts of invertebrate predators, but may not be effective against vertebrates, this study, furthermore, investigated whether the exposure to a vertebrate predator (i.e., the sunbleak *Leucaspius delineatus*) does induce an increased spinescence. A lack of such a response to a vertebrate predator could further indicate that the alteration in spinescence is not a general response to predator stress.

## 2 MATERIALS AND METHODS

### 2.1 Organisms

Our experiments were conducted using the *D*. *magna* clone K_3_4J originating from a former fishpond near Ismaning, Germany (Rabus & Laforsch, 2011), and a *D. longicephala* clone originating from Lara Pond in southeast Australia (Hebert, 1977; Kruppert et al., 2016; Weiss et al., 2015), both of which are cultured at the Department of Animal Ecology 1 (University of Bayreuth, Germany). The invertebrate predators we used were the tadpole shrimp *T*. *cancriformis* cultured at the Department of Animal Ecology 1 (University of Bayreuth), originally provided by Dr. Erich Eder (University of Vienna), and the backswimmer *N. maculata*, collected from outdoor concrete basins at the University of Bayreuth. The vertebrate predator used in our experiments was the small cyprinid sunbleak (*L. delineatus*) also cultured at the Department of Animal Ecology 1 (University of Bayreuth).

### 2.2 Exposure to predator kairomones (induction experiments)

Induction experiments were conducted in a climate chamber, at a constant temperature of 20 °C ± 0.5 °C under fluorescent light. The light–dark regime was 15:9 h, with 30 min of increasing light and decreasing light, respectively, at the beginning and at the end of the light period, for dawn or dusk. For maximum, early on, one‐generation induction, the experimental animals were exposed to the predator cues or reared without a predator for the control, as part of the third clutch of their mothers, that is, during their embryonic stages, as soon as they had been released into the brood pouch. This embryonic predator exposure was conducted in 2 L glass beakers (LABSOLUTE, Th. Geyer GmbH and CO. KG.), one beaker per treatment, with each beaker containing 15–20 individuals of the tested *Daphnia* species. The daphnids were held in semi‐artificial medium (SSS‐medium (Jeschke & Tollrian, 2000)), based on well water, ultrapure water, salt, phosphate buffer, and trace elements, with additional 20 ml CaCl_2_ to correct for the low calcium level in our regional water. For the invertebrate predator embryonic and postnatal predator exposure of morphological defenses in *D. magna*, *T*. *cancriformis* (2–2.5 cm body length) were directly introduced into the respective treatment replicates, by hanging net cages, each containing one predator, into the experimental 2 L beakers. To test for the induction of morphological defenses by an invertebrate predator in *D. longicephala*, this predator exposure setup was repeated, only with adult *N. maculata* (one per cage). The cylindrical net cages containing the predators were made of an acrylic glass frame (length 13.5 cm, diameter 7.5 cm, thickness 3 mm) with windows (5 cm width, 6.5 cm length) as well as an open floor, all of which were covered with gauze (nylon, mesh size 160 μm), separating the predator from the prey while ensuring the exchange of kairomones through the nylon mesh. The predators were each fed five larvae of *Chironomus* sp. and ten adult daphnids of the respectively induced *Daphnia* species daily, which further ensured a thorough induction through the production of alarm cues (Laforsch et al., 2006). The exchange of kairomones and alarm cues with the surrounding medium was ensured through manually moving the cages, in a gentle, vertical movement twice a day. For the control treatments in the *D*. *magna* and *D. longicephala* experiments, one beaker in each experiment containing SSS‐medium was also supplied with one net cage each. These net cages contained the same daily food as in the predator treatments and were moved twice daily, as well, to exclude any effects on the prey organisms’ induction deriving from the cage, the predators’ food, or the created water turbulence through the manual cage movement. The medium in all beakers was exchanged every other day, and thereby, all net cages were thoroughly cleaned from biofilm. *Daphnia* spp. were fed *ad libitum* with *Acutodesmus obliquus* daily. In contrast to the invertebrate treatment, the workflow for the vertebrate treatment had to be adapted to these rather large animals, to create as little stress for the used *L. delineatus* as possible. Therefore, in difference to the control and invertebrate predator treatment, the fish treatment had to be prepared in a larger medium while the handling of these animals in general was reduced to the necessary minimum. Thus, the *Leucaspius*‐induction medium for the embryonic and postnatal predator exposure was prepared by rearing one *L. delineatus* in 5 L SSS‐medium for 24 h, at the same food conditions as the invertebrate predators. Afterward, the utilized *L. delineatus* was removed and the produced *Leucaspius* medium filtered through a fluted filter (Type 589/1 1/2, Schleicher & Schüll GmbH), with 12–25 μm particle retention, to remove large organic particles. The *Leucaspius* medium, in which the experimental daphnids of this treatment were placed after preparation, was exchanged daily to ensure a constant kairomone concentration in the medium (De Meester & Cousyn, 1997; Tollrian, 1993). For the preparation of the *Leucaspius* medium, the *L. delineatus* was exchanged daily to minimize individual stress for these predators.

In the next step, within each of the two experiments, the same‐aged embryonically predator‐exposed neonates of the third clutch were randomly assigned to the postnatal predator exposure replicates, of their respective treatment. In the experiment with *D*. *magna* as well as in that with *D. longicephala*, the respective invertebrate predator treatments and the control treatments comprised 15 2 L beakers per treatment, containing 13 to 14 randomly assigned neonates per beaker, the beakers containing either amount of daphnids equally distributed among treatments. The beakers were filled with 2 L of SSS‐medium and one of the previously described net cages was placed into each beaker. These beakers for the treatment of the respective neonates contained the same predators, or none, and food as described for the embryonic predator exposure, respectively, for each treatment. For the *Leucaspius* treatment in both, the experiment with *D. longicephala* and the experiment with *D*. *magna*, same‐aged neonates of the respective *Daphina* species, which were embryonically predator‐exposed with *Leucaspius* medium, were randomly assigned to 160 ml WECK glasses (J. WECK GmbH & Co. KG), one neonate each. These glasses contained 100 ml *Leucaspius* medium, which was prepared as described for the embryonic predator exposure. For this treatment in both experiments, the amount of medium was reduced to keep the number of *L. delineatus*, necessary for the kairomone medium production, also as low as possible. For each of the two *Daphnia* experiments, the *Leucaspius* treatment was replicated 30 times. The experiments were terminated when the individuals of *D*. *magna* and *D. longicephala* reached their age of first reproduction. The animals were preserved in 70% ethanol p.a. (Rabus & Laforsch, 2011) and stored in the refrigerator at 1 °C until further analysis.

### 2.3 Measurements

**FIGURE 1:**
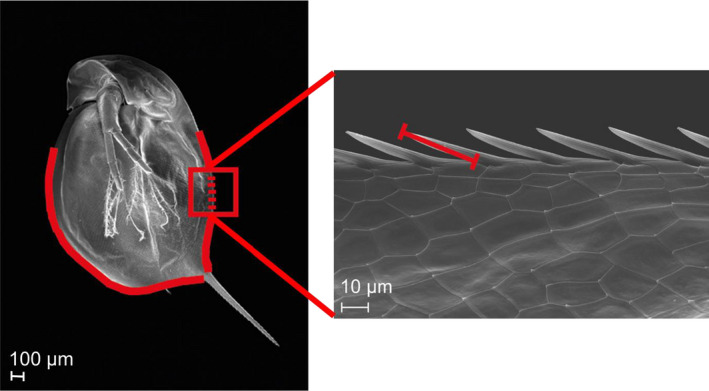
Location and approximate extension of the dorsal and ventral SBA in *D*. *magna* and the exemplified location and character of a spinule measurement as conducted in our study

Pictures of *D*. *magna* and *D. longicephala* were taken using a digital camera (DP26, OLYMPUS Deutschland GmbH) attached to a stereomicroscope (Leica M 50, Leica MiKrosysteme Vertrieb GmbH). All recorded morphological traits were measured using a digital image analysis software (cellSens Dimension 1.11; OLYMPUS Deutschland GmbH). In the invertebrate and the control treatment of each experiment, three animals of each replicate were selected randomly and measured. In the *Leucaspius* treatment of each experiment, 15 randomly chosen individuals were measured to match the replicate number of the two other treatments. We measured the tail‐spine length, the crest size (for *D. longicephala* only), the length of the dorsal and ventral SBA, and the length of the dorsal and ventral spinules. The tail‐spine length was defined as the distance from the base of the tail‐spine to its tip. In *D. longicephala*, the crest size was measured from the rostral side of the compound eye to the most distant point at the dorsal crest/head expansion. On all animals, we measured the length of the dorsal and ventral spinules‐bearing area, along the row of protruding spinules at the dorsal and ventral ridges of the carapace (Figure 1). To compensate for size‐dependent effects, we calculated the relative values for these traits. For this calculation, we measured the body length of each animal from the top of the compound eye to the ventral base of the tail‐spine. Afterward, relative values of the respective trait in relation to each animal's body length in percent were calculated. Finally, we calculated the mean length of the dorsal and ventral spinules. For this calculation, the lengths of the five spinules in the middle of the SBAs were measured, from inner base of the spinules to each respective tip (Figure 1).

### 2.4 Statistical analysis

The statistical analysis was carried out for each species and trait separately using R (version 4.0.2; R Core Team, 2020) and RStudio (version 1.1.453; RStudio Team, 2019) with the appropriate packages: emmeans (Lenth, 2020), FSA (Ogle et al., 2020), lmerTest (Kuznetsova et al., 2017), and car (Fox & Weisberg, 2019). All graphs were obtained with ggplot (Wickham, 2016) and ggsignif (Ahlmann‐Eltze, 2019).

The data were, firstly, analyzed using a linear model (LM) and a nested linear mixed model (LMM). For the LMM, the respective morphological trait was used as fixed effect, while the glass/rearing was the nested random effect, since the animals for the *Leucaspius* treatment had to be reared differently than the ones in the control and in the *Triops* treatment. This was followed by a trait‐wise comparison of the models for fit, via a log‐likelihood analysis (LH). In cases where LMM was the significantly better fit, the glass/rearing proved to have impacted the data of the respective trait, and the LMM was used for further analysis to exclude this impact for interference. In the cases where the LMM was not a significantly better fit the LM was used. Using a Shapiro–Wilk test and a QQ‐Plot with confidence intervals, the residuals of the model, concerning each trait individually, were checked for normal distribution. Additionally, we conducted a Levene test to check for homogeneity of variance of the model's residuals. In cases where the assumptions were met, a type two ANOVA, with a consecutive Tukey HSD post hoc test was performed with a Holm correction for multiple comparison. Where the assumptions of the ANOVA were violated, we conducted a Kruskal–Wallis test, followed by a Dunn's post hoc test with a Holm correction for multiple comparison.

## 3 RESULTS

### 3.1 *D. magna* experiment

**FIGURE 2:**
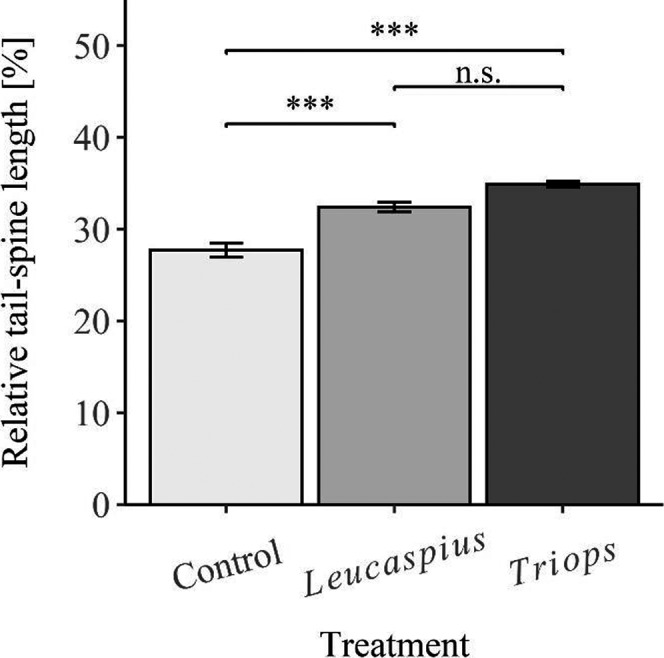
Relative tail‐spine length of *D*. *magna* exposed to a vertebrate (*L*. *delineatus*), an invertebrate predator (*T. cancriformis*), and the control (asterisks indicate levels of significance: * <0.05, ** <0.01, *** <0.001; n.s. = no statistically significant differences; error bars represent standard error)

**FIGURE 3:**
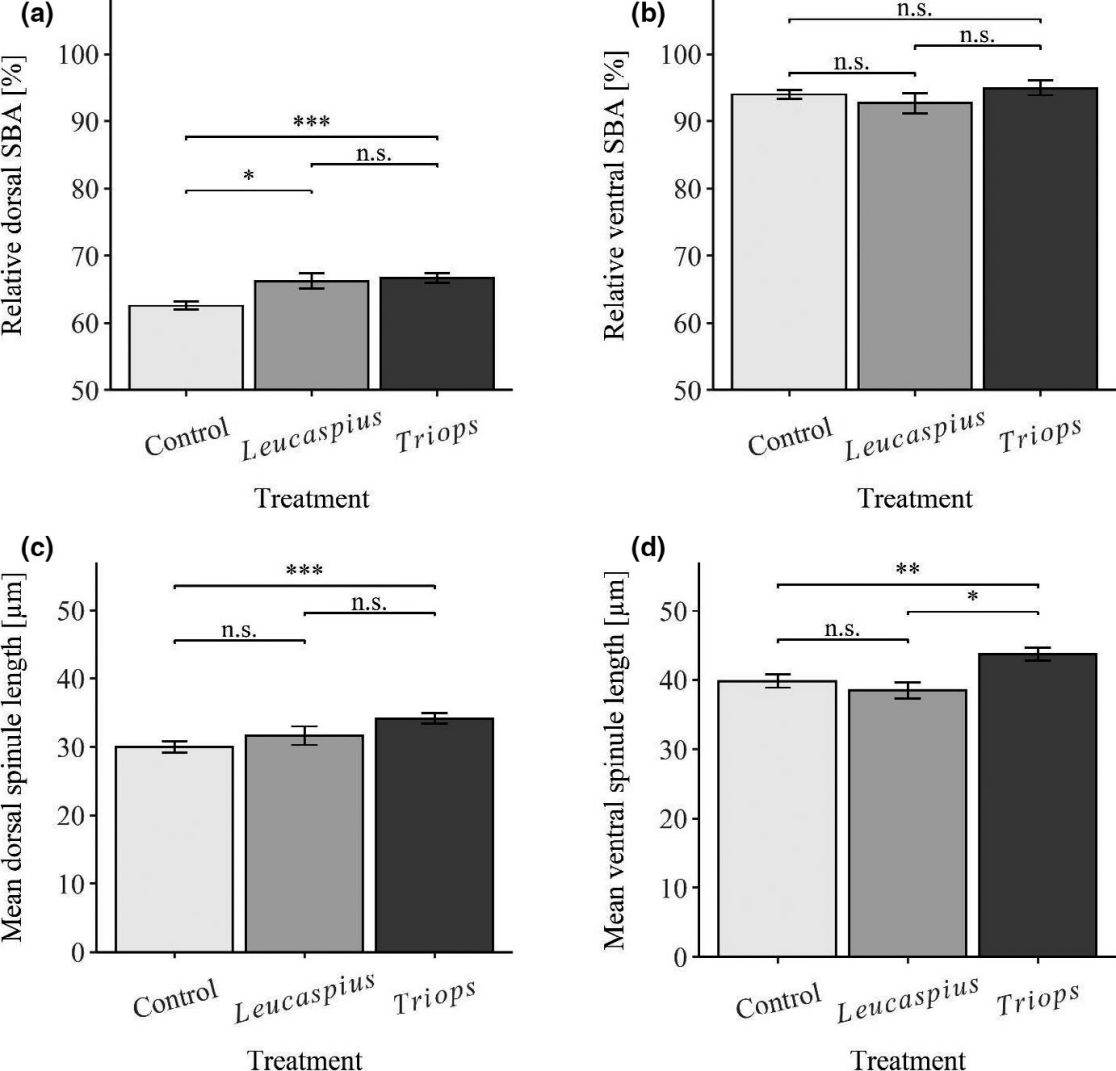
Comparison of dorsal and ventral spinules‐bearing areas (SBAs) and mean spinule lengths of *D*. *magna* exposed to a vertebrate (*L*. *delineatus*), an invertebrate predator (*T. cancriformis*), and the control (asterisks indicate levels of significance: * <0.05, ** <0.01, *** <0.001; n.s. = no statistically significant differences; error bars represent standard error)

Individuals from the three treatments differed significantly in their relative tail‐spine length (ANOVA; *F*_2, 100_ = 21.651; *p* < .001). The relative tail‐spine length of control individuals was significantly smaller than that of *Leucaspius*‐exposed individuals (Tukey's HSD; *p* = .001; Figure 2) and *Triops*‐exposed individuals (Tukey's HSD; *p* < .001; Figure 2), whereas there was no significant difference between *Leucaspius*‐ and *Triops*‐exposed individuals (Tukey's HSD; *p* = .111; Figure 2). Similarly, the individuals of the three treatments differed significantly in the relative length of their dorsal SBA (Kruskal–Wallis test; chi‐sq = 23.78; df = 2; *p* < .001). Here, control animals had significantly smaller relative dorsal SBAs than *Leucaspius*‐induced (Dunn's; *p* = .016; Figure 3a) and *Triops*‐induced individuals (Dunn's; *p* < .001; Figure 3a). Furthermore, there was no significant difference between *Triops*‐exposed and *Leucaspius*‐exposed animals (Dunn's; *p* = .476; Figure 3a). There was no significant difference in the relative length of the ventral SBA across the treatments (ANOVA; *F*_2, 102_ = 0.818; *p* = .444; Figure 3b). Despite this, animals of the three treatments significantly differed in the mean length of the dorsal spinules (ANOVA; *F*_2, 102_ = 7.0177; *p* = .001). In this, the mean length of the dorsal spinules of *Triops*‐exposed individuals was significantly larger than that of control individuals (Tukey's HSD; *p* < .001; Figure 3c), while there were no significant differences either between *Leucaspius*‐ and *Triops*‐exposed (Tukey's HSD; *p* = .256; Figure 3c), or between *Leucaspius*‐exposed and control individuals (Tukey's HSD; *p* = .544; Figure 3c). Individuals of the three treatments differed significantly in the mean length of the ventral spinules (ANOVA; *F*_2, 102_ = 6.3649; *p* = .002). The mean length of the ventral spinules in *Triops*‐exposed animals was thereby significantly larger than in control (Tukey's HSD; *p* = .009; Figure 3d) or in *Leucaspius*‐exposed individuals (Tukey's HSD; *p* = .014; Figure 3d). However, there was no statistically significant difference between control and *Leucaspius*‐exposed individuals (Tukey's HSD; *p* = .741; Figure 3d).

### 3.2 *D. longicephala* experiment

**FIGURE 4:**
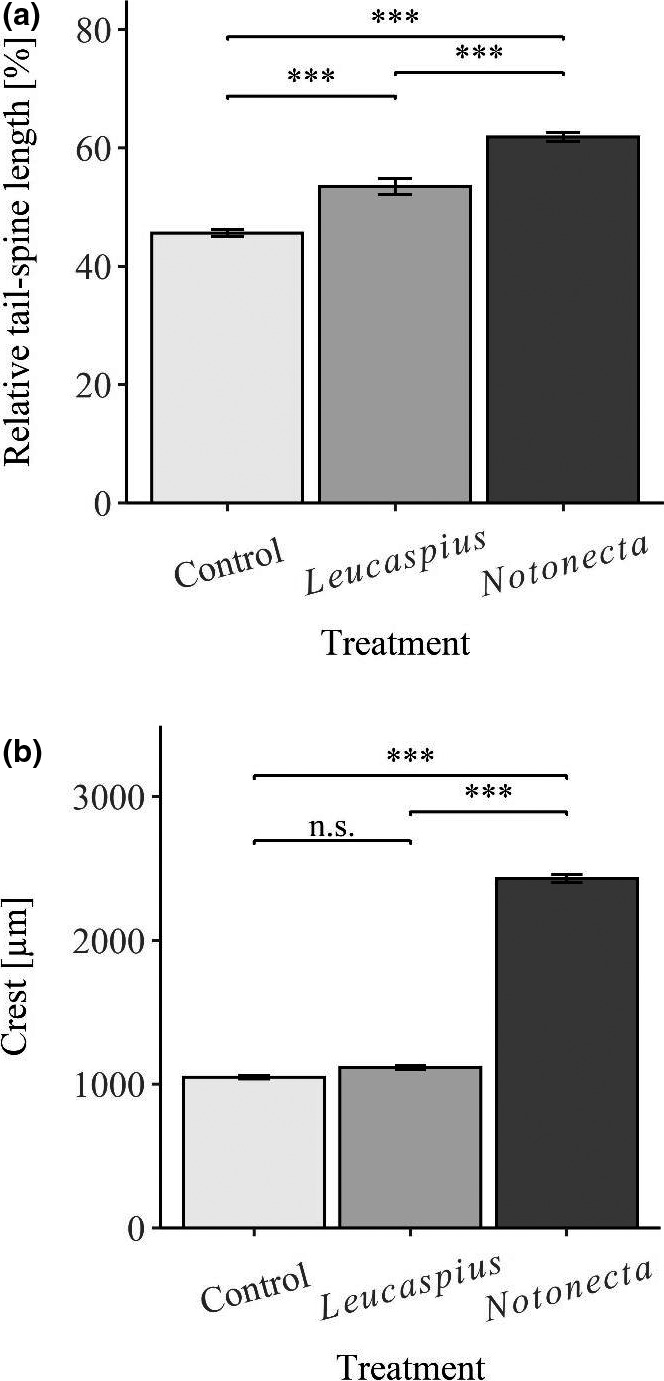
Comparisons of the relative tail‐spine and crest‐induction of *D. longicephala* exposed to a vertebrate (*L*. *delineatus*), an invertebrate predator (*N*. *maculata*), and the control (asterisks indicate levels of significance: * <0.05, ** <0.01, *** <0.001; n.s. = no statistically significant differences; error bars represent standard error)

**FIGURE 5:**
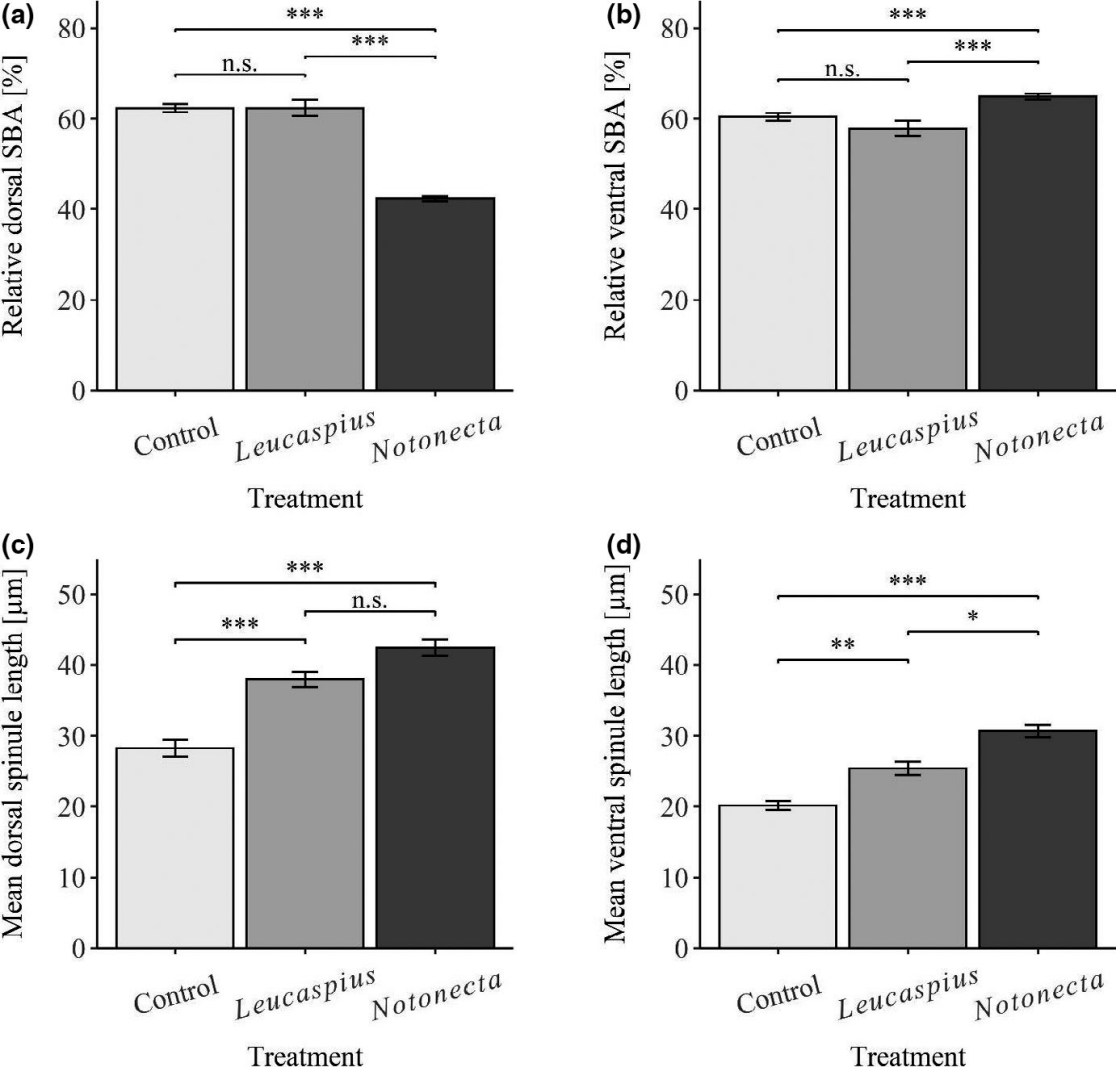
Comparison of dorsal and ventral spinules‐bearing areas (SBAs) and mean spinule lengths of *D. longicephala* exposed to a vertebrate (*L*. *delineatus*), an invertebrate predator (*N*. *maculata*), and the control (asterisks indicate levels of significance: * <0.05, ** <0.01, *** <0.001; n.s. = no statistically significant differences; error bars represent standard error)

Individuals of the three treatments differed significantly in their relative tail‐spine length (ANOVA; *F*_2, 85_ = 132.94; *p* < .001). The relative tail‐spine length of *Notonecta*‐exposed daphnids was significantly longer than that of the control (Tukey's HSD; *p* < .001; Figure 4a) and *Leucaspius*‐exposed animals (Tukey's HSD; *p* < .001; Figure 4a). Moreover, the relative tail‐spine length of the control individuals was significantly smaller than that of *Leucaspius*‐exposed individuals (Tukey's HSD; *p* < .001; Figure 4a). The animals of the three treatments differed significantly in their crest size (Kruskal–Wallis test; chi‐sq = 76.99; df = 2; *p* < .001). *Notonecta*‐exposed individuals developed significantly larger dorsal head extensions (i.e., crests) compared to the control (Dunn's; *p* < .001; Figure 4b) and *Leucaspius*‐induced (Dunn's; *p* < .001; Figure 4b) animals. In contrast, there was no significant difference between control and *Leucaspius*‐induced animals concerning the crest (Dunn's; *p* = .051; Figure 4b). Concerning the relative length of the dorsal SBA, individuals of the three treatments also differed significantly (Kruskal–Wallis test; chi‐sq = 67.64; df = 2; *p* < .001). In this, the relative dorsal SBA of *Notonecta*‐exposed individuals was significantly smaller than that of *Leucaspius*‐exposed animals (Dunn's; *p* < .001; Figure 5a) or control individuals (Dunn's; *p* < .001; Figure 5a). However, the relative length of the dorsal SBA of *Leucaspius*‐exposed animals did not differ significantly from that of control individuals (Dunn's; *p* = .568; Figure 5a). The individuals of the three treatments differed significantly in the relative length of the ventral SBA (Kruskal–Wallis test; chi‐sq = 27.47; df = 2; *p* < .001). The relative ventral SBA of *Notonecta*‐induced individuals was significantly larger than of *Leucaspius*‐exposed (Dunn's; *p* < .001; Figure 5b) and control animals (Dunn's; *p* < .001; Figure 5b). Furthermore, the relative length of the SBA of control individuals did not differ significantly from that of *Leucaspius*‐exposed animals (Dunn's; *p* = .243; Figure 5b). Additionally, the animals of the three treatments differed significantly in the mean length of the dorsal spinules (ANOVA; *F*_2, 98_ = 40.835; *p* < .001). Thereby, the mean length of the dorsal spinules in control animals was significantly smaller than in *Leucaspius*‐exposed (Tukey's HSD; *p* < .001; Figure 5c) and in *Notonecta*‐exposed individuals (Tukey's HSD; *p* < .001; Figure 5c). However, the mean length of dorsal spinules of *Notonecta*‐induced individuals was not significantly different from that of *Leucaspius*‐exposed individuals (Tukey's HSD; *p* = .112; Figure 5c). Despite this, the animals of the three treatments differed significantly in the mean length of the ventral spinules (Kruskal–Wallis test; chi‐sq = 55.59; df = 2; *p* < .001). The mean length of the ventral spinules of *Notonecta*‐exposed individuals was significantly larger than of control individuals (Dunn's; *p* < .001; Figure 5d) or *Leucaspius*‐exposed animals (Dunn's; *p* = .014; Figure 5d). Moreover, the mean length of the ventral spinules was significantly larger in *Leucaspius*‐exposed individuals than in control individuals (Dunn's; *p* = .007; Figure 5d).

## 4 DISCUSSION

For the here tested *Daphnia* species, *D*. *magna* and *D. longicephala*, previous studies demonstrated the expression and effectiveness of large‐scale inducible morphological defenses (Barry, 2000; Rabus & Laforsch, 2011; Rabus et al., 2012; Weiss et al., 2015). Hence, the relative tail‐spine lengths of each species, as well as the crest for *D. longicephala*, were used as indicators, to see whether both species responded to the respective predator cues. In accordance with previous studies, both species developed elongated tail‐spines, and *D. longicephala* an additional crest, when exposed to their respective invertebrate predators. Rabus et al. (2012) previously demonstrated the increased survival rate of defended *D*. *magna* compared to the undefended morph under predation threat by the gape limited predator *T*. *cancriformis* of similar size as we used them in this experiment. Though the exact mode of action for the induced large‐scale defenses of *D. longicephala* against *Notonecta* sp. has not been demonstrated, yet these defenses are thought to change the hydromechanical swimming/escape efficiency of the animal (Hebert, 1978; O’Brien & Vinyard, 1978) and/or create handling difficulties for this predator (Dodson, 1974; Jeschke et al., 2008; Kruppert et al., 2017). Both *Daphnia* species profit from a longer handling time, since chances for the animals to escape their predators increase simultaneously. Our results confirm the defense expression of *D*. *magna* and *D. longicephala* in response to their invertebrate predators through an enlargement of the measured large‐scale defenses, as predicted.

In response to fish kairomones, we expected an increase in the relative tail‐spine length of *D*. *magna* and *D. longicephala*, since for some daphnids, enlarged spines were previously shown to be rather beneficial defenses against fish (Engel et al., 2014; Swaffar & O’Brien, 1996). One study indicating a defensive function of the tail‐spine elongation in response to fish features daphnids, belonging to the *D. galeata* complex (Spaak & Boersma, 1997). Furthermore, we expected no development of a crest in *D. longicephala*, as this defense has so far only been expressed in response to predators of the family Notonectidae (Barry, 2000; Barry & Bayly, 1985; Grant & Bayly, 1981; Weiss et al., 2015). In concordance with the study mentioned above (Spaak & Boersma, 1997), our results show that the fish‐exposed *D*. *magna* and *D. longicephala* clone develops a significantly longer relative tail‐spine than control animals, while *D. longicephala*, additionally, did not develop a crest, thus most likely confirming a sufficient induction in response to this vertebrate predator. However, it has to be noted that only a single clone was used to represent each species and clonal differences can apply to both large‐scale and small‐scale morphological alterations in response to predator kairomones (Boeing et al., 2006; Juračka et al., 2011; Rabus et al., 2012; Riessen, 2012).

While the benefit of the abovementioned predator‐induced large‐scale defenses of *Daphnia* has been shown in many cases (Diel et al., 2020), there is a lack of knowledge regarding alterations of small‐scale structural changes, such as spinules, in response to predator cues. In some *Daphnia*, for example, *D. barbata* and *D*. *similis*, morphological changes of spinescence in response to predator cues were already discussed as an inducible morphological defensive trait (Fryer, 1991; Hebert & Grewe, 1985; Herzog & Laforsch, 2013; Ritschar et al., 2020).

Given that the expression of spines is a frequently utilized defense structure in nature (Inbar & Lev‐Yadun, 2005), the elongation of spinules and SBAs in the clones of both predator‐exposed *Daphnia* species appears as a defense. However, it must be considered that *D*. *magna* and other *Daphnia* species change their entire shape in reaction to predators (Herzog & Laforsch, 2013; Rabus & Laforsch, 2011; Ritschar et al., 2020), which might involve carapace modifications, such as an alteration of the SBAs. Furthermore, ultrastructural defensive strengthening of *D. magna*’s and *D. longicephala*’s carapace has been found in reaction to the here used predators, even for the same *Daphnia* clones as we used in our experiments (Kruppert et al., 2017; Rabus et al., 2013). One may speculate that the elongation of the spinules in response to predators may be connected to the overall modification of the carapace, without them having an autonomous defensive function. As changes in the ultrastructure and the shape of the entire carapace were not included in our study, we cannot confirm nor deny this hypothesis. Hence, it cannot be excluded that alterations in spinescence are developmentally connected to changes of more conspicuous defensive traits in general. However, to isolate the protective effect of a single trait of inducible morphological defenses in daphnids is hardly possible. For instance, *D. cucullata* develops a fortified carapace and an elongated helmet in response to various invertebrate predators (Laforsch & Tollrian, 2004b; Tollrian, 1990). It is not possible to test whether the helmet or the fortified carapace is responsible for the protective effect since one trait is accompanied by the other and a manipulation of the traits is impossible.

However, it has already been shown that *D. barbata* responds to different predatory invertebrates with different arrays of morphological inducible defenses including alterations in spinescence (Herzog & Laforsch, 2013). Based on the “concept of modality” of predators, it has been discussed that evolution can favor these specialized defenses over a general defense. This implies that, also, inconspicuous alterations, such as the angle and the length of the spinules, can contribute to the overall defense. In *D. barbata*, for instance, the angle and length of the spinules differed considerably not only between control‐treatment daphnids and predator‐exposed ones (which may indicate that alterations in spinescence are developmentally connected to changes in body shape), but also between *Notonect*a sp. and *T*. *cancriformis*‐exposed *D. barbata*, indicating that spinules are inducible morphological defensive traits, adapted to the respective predator regime. Therefore, spinules may also contribute to the fine‐tuning of the phenotype to be best protected against a specific predator that has a specific catching apparatus or handling pattern. Further, in *T*. *cancriformis*‐exposed *D. atkinsoni*, the so‐called crown of thorns, a dorsal carapace extension that covers the head and features spinules along their rims, has been shown to increase the survival under predation threat by this predator (Laforsch et al., 2009; Petrusek et al., 2009). Nonpredator‐exposed daphnids only display the dorsal carapace extension without any spinules, strongly indicating that also spinules likely are inducible defensive traits in *Daphnia* and not generally by‐products of an alteration in body shape. The effectivity of the inducible morphological defense in *D. atkinsoni* is suspected to be the combined result of some inference of the “crown's” spinules, the elongated tail‐spine, and potentially an enhanced ultrastructure of the carapace with the feeding apparatus of *T*. *cancriformis* (Laforsch et al., 2009).

In a similar manner, the predator‐specific spinule elongation along the carapace rims of *T*. *cancriformis*‐exposed *D*. *magna*, that was discovered in our study, might add to the protective effect of the morphological large‐scale (tail‐spine, Rabus et al., 2012) and ultrastructural defenses (carapace strength, Rabus et al., 2013) of this clone, by increasing the handling time through *T*. *cancriformis*. After catching the prey, *T*. *cancriformis* moves it toward the midventral food groove, through it, and to the mouth where, using its mandibles, food is cut into pieces of a manageable size and ingested (Fryer, 1988; Rabus & Laforsch, 2011). Prior to being cut into pieces, however, the prey is passed through a series of anterior trunk limbs with gnathobases, along which spikes can be found (Fryer, 1988). Furthermore, the food groove shows sweeping spines on the endites of the endopods, and cleaning bristles along the food grooves lateral margins (Fryer, 1988). To transport prey in the midventral food groove, *T*. *cancriformis* were observed to turn the prey (Fryer, 1988) and in case of *D*. *magna* subjectively turn prey items rather often so that their dorsal carapace ridge faced the gnathobases (Rabus, personal communication). Here, *D. magna*'s longer dorsal spinules may cause the *Triops*’ gnathobases to get caught with their spikes. Further, *D. magna*'s elongated dorsal and ventral spinules may also get entangled in the predator's cleaning bristles along the food grove, or in the sweeping spines of *T*. *cancriformis*’ endopods. (Fryer, 1988). The resulting increased handling time, potentially offers morphologically defended *D*. *magna* more chances for escaping their invertebrate predator's grasp.

In contrast to the feeding mechanism of *T*. *cancriformis*, smaller fish, like *L. delineatus*, usually suck their prey into the mouth cavity and either crush it with their pharyngeal teeth prior to ingestion, for example, cyprinids, or swallow it whole (Lauder, 1980; Muller et al., 1982). Therefore, an investment in small‐scale defenses like spinule‐elongation seems nonbeneficial for fish‐exposed daphnids, as *L. delineatus* possesses no fine structures in its mouth to get entangled in. Similarly, our results show that the spinules of *D*. *magna* of the *L. delineatus* treatment remain the same length as found in the control, indicating that plasticity in spinescence of this species could be a predator‐specific adaptation.

*D. magna*'s predator‐specific (invertebrate vs. vertebrate) changes of the spinule length are accompanied by a different, pattern concerning the SBAs across both predator treatments. In this, the dorsal SBA was generally elongated while the ventral SBA generally showed no significant difference to the control across both, the invertebrate and the vertebrate, predator treatments. In previous studies, *Daphnia*'s alarm signals, communicating the wounding of conspecifics were found to induce morphological defenses, to a certain degree (Laforsch et al., 2006; Pestana et al., 2013). As the dorsal SBA of *D*. *magna* was enlarged under both predation regimes, we suggest that this trait could be part of a rather general defense, possibly in reaction to alarm cues, which only signal a predation threat in close proximity but not the specifics of the predator. While these small‐scale traits appear in a morphologically connected manner, another possible explanation for the difference in expressed patterns, between mean spinule lengths and relative SBAs in *D*. *magna*, is the possibility of the SBA expression differing in developmentally associated origin from the spinule lengths. While the spinule lengths, expressed by *D*. *magna* in our experiment, may be associated with an autonomous defensive benefit, the SBAs might be a by‐product of other expressed predator‐induced traits. Furthermore, the fish treatment setup had to differ from the other two treatment setups for practical and animal‐welfare reasons. Therefore, we cannot exclude that the differing setup features between the invertebrate/control setups and the vertebrate setup (e.g., medium exchange regime, presence/absence of cage and its movements) may alter the response of the daphnids.

**FIGURE 6:**
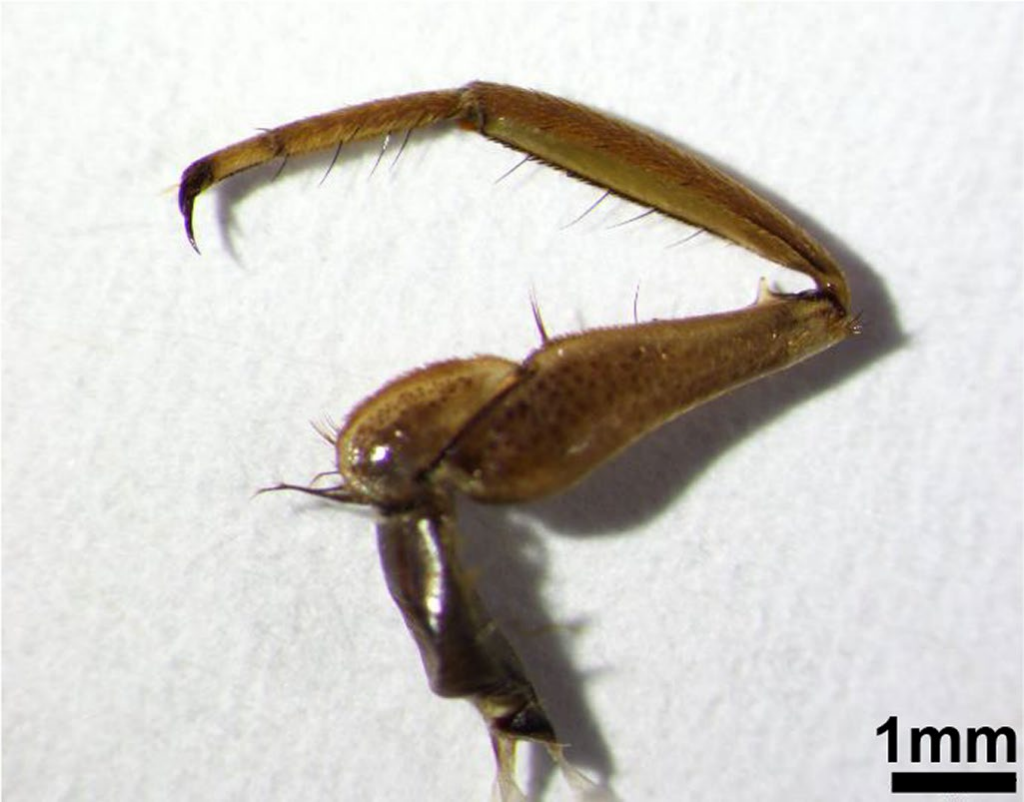
One of four anterior legs of *N*. *maculata*. Short hairs along the inner face are interrupted by longer hairs. Very short hairs cover the whole leg

The patterns of the here discovered small‐scale changes also differed between *D*. *magna* and *D. longicephala*. In *D. longicephala*, both SBAs showed no length changes in reaction to the vertebrate treatment. This was expected, as there is no indication of feeding structures in *L. delineatus*, which could be impeded by small‐scale defenses in *Daphnia* during prey ingestion or handling, as pointed out above for *D*. *magna*. On the other hand, the exposure to the invertebrate *N. maculata* leads to an elongation of the ventral SBA and a significantly downsized dorsal SBA. An enlarged area presenting spinules along the carapace of *N. maculata*‐exposed *D. longicephala* could potentially contribute a defensive benefit in connection with the handling and feeding process of *N. maculata*. Triggered by mechanical and visual cues, notonectids catch their prey with their four anterior legs, grip them in a hug‐like manner and sting them with their proboscis before digesting them externally and imbibing the product of that digestion (personal observation (Dahm, 1972)). The inner face of its four raptorial anterior legs bears a line of short hairs, regularly interrupted by longer, bristle‐like hairs (Figure 6; personal observation, (Gittelman, 1977)). Additionally, these legs are covered with very short hairs all over. We propose that the enlarged ventral SBA of *N. maculata*‐exposed *D. longicephala* could potentially interfere with some structures on the four anterior legs of this invertebrate predator. This interference by spinules may be similar to the interference of neckteeth in *D. pulex* with the catching apparatus of *Chaoborus* larvae (e.g., Tollrian, 1995) or for *T*. *cancriformis*’ prey‐capturing and handling of respectively “crowned” *D. atkinsoni* (Petrusek et al., 2009). The contrasting dorsal SBA reduction found in our study we suggest to be the result of a trade‐off in favor of the induced crest developed by *Notonecta* sp.‐exposed *D. longicephala* (Barry, 2000; Grant & Bayly, 1981; Laforsch et al., 2006). The inducible defensive crest considerably extends not only lateral–dorsally, but also caudally along the dorsal ridge and thereby potentially limits the space available for the expression of spinules.

In contrast to these potentially predator‐specific small‐scale morphological changes of *D. longicephala*, that is, the SBAs, the spinule length of *D. longicephala* increased after exposure to both, *L. delineatus* and *N. maculata*, compared to the control. However, in *N. maculata*‐exposed *D. longicephala*, spinule length was more pronounced compared to the *L. delineatus* treatment. A possible reason for this morphological change is that the elongation of the spinules in *N. maculata*‐exposed *D. longicephala* may interfere with the feeding process of the invertebrate predator, as outlined above for the SBA. Despite this, we, primarily, do not anticipate a distinct advantage of any elongated spinules of *D. longicephala* in predatory contact with *L. delineatus*, for the same reasons as discussed for *D*. *magna*. Therefore, as discussed above with respect to *D*. *magna*, we suggest that the spinule elongation may either be developed as a rather general defense against predation, or as a by‐product of another predator‐induced morphological trait. A previous study modelling the functional defensive contribution of structural and shape alterations in predator‐induced *D. longicephala*, using finite element analysis, found these two kinds of defenses to differ in their contribution to the defense (Kruppert et al., 2017). In this, structural alterations were found to contribute to carapace resistance, while shape alterations do not, but are rather likely to impede catching or handling through *Notonecta* sp. On the basis of this functionally differing purpose of defensive shape and structure changes, the by‐product character of predator‐induced spinule length changes, that is, shape changes, in *D. longicephala*, if true, would not be connected to ultrastructural, but to other shape alterations. As a result, shape alterations could serve the same functional purpose and, therefore, could also originate from a related origin. Despite this, overall, the different patterns of the predator‐induced changes of spinescence in *D. longicephala* and *D*. *magna* indicate the addition of small‐scale changes to the wide array of predator‐specific induced large and ultrastructural defenses of each respective clone.

Our study is a first indication of the specificity of small‐scale predator‐induced morphological changes expressed by *D*. *magna* and *D. longicephala*. This level of phenotypic plasticity is thereby suggested to add to the respective predator‐specific defenses. Our results, moreover, suggest that invertebrate predator exposure might result in a general elongation of the spinules, which varies species‐specifically in its expressed magnitude, as shown before for *D. ambigua* (Hebert & Grewe, 1985), *D. barbata* (Herzog & Laforsch, 2013), and *D*. *similis* (Ritschar et al., 2020). In difference to this similarity, the tested *Daphnia* clones showed diverging reactions to vertebrate predator exposure in our experiments. The combination of morphological small‐scale predator‐induced changes with the respective large‐scale defenses, that is, crest development and/or tail‐spine elongation (Laforsch et al., 2006; Rabus & Laforsch, 2011), and ultrastructural defenses (Kruppert et al., 2017; Rabus et al., 2013) may comprise an effective set of defensive traits, specifically adapted to a certain predation risk. However, a different explanation for some of the here found changes could be a difference in treatment setup, as well as a connected origin of these defenses with other predator‐induced morphological traits. Nevertheless, the discovery of the variety of induced morphological small‐scale changes demonstrates the complexity of phenotypic plasticity in defensive traits, which carries potential for future basic research into the functionality of the entirety of predator‐induced morphological changes in *Daphnia*.

## CONFLICT OF INTEREST

None declared.

## AUTHOR CONTRIBUTIONS

**Patricia Diel:** Conceptualization (equal); Formal analysis (lead); Investigation (lead); Methodology (equal); Project administration (equal); Software (lead); Validation (equal); Visualization (lead); Writing‐original draft (equal); Writing‐review & editing (equal). **Max Rabus:** Conceptualization (equal); Formal analysis (supporting); Methodology (equal); Project administration (equal); Validation (equal); Visualization (supporting); Writing‐original draft (equal); Writing‐review & editing (equal). **Christian Laforsch:** Conceptualization (equal); Methodology (equal); Project administration (equal); Resources (lead); Supervision (lead); Validation (equal); Visualization (supporting); Writing‐original draft (equal); Writing‐review & editing (equal).

## Data Availability

Data acquired with and used for the analysis of the experiments in this study are available on (Dryad, https://doi.org/10.5061/dryad.jwstqjq9n).
